# Antibiotic Resistance Gene Abundances Correlate with Metal and Geochemical Conditions in Archived Scottish Soils

**DOI:** 10.1371/journal.pone.0027300

**Published:** 2011-11-09

**Authors:** Charles W. Knapp, Seánín M. McCluskey, Brajesh K. Singh, Colin D. Campbell, Gordon Hudson, David W. Graham

**Affiliations:** 1 Department of Civil Engineering, David Livingstone Centre for Sustainability, University of Strathclyde, Glasgow, United Kingdom; 2 Hawkesbury Institute for the Environment, University of Western Sydney, Penrith, Australia; 3 The James Hutton Institute, Aberdeen, United Kingdom; 4 Department of Soil and Environment, Swedish University of Agricultural Sciences, Uppsala, Sweden; 5 School of Civil Engineering and Geosciences, Newcastle University, Newcastle upon Tyne, United Kingdom; Argonne National Laboratory, United States of America

## Abstract

The vast majority of antibiotic resistant genes (ARG) acquired by human pathogens have originated from the natural environment. Therefore, understanding factors that influence intrinsic levels of ARG in the environment could be epidemiologically significant. The selection for metal resistance often promotes AR in exposed organisms; however, the relationship between metal levels in nature and the intrinsic presence of ARG has not been fully assessed. Here, we quantified, using qPCR, the abundance of eleven ARG and compared their levels with geochemical conditions in randomly selected soils from a Scottish archive. Many ARG positively correlated with soil copper levels, with approximately half being highly significant (*p*<0.05); whereas chromium, nickel, lead, and iron also significantly correlated with specific ARG. Results show that geochemical metal conditions innately influence the potential for AR in soil. We suggest soil geochemical data might be used to estimate baseline gene presence on local, regional and global scales within epidemiological risk studies related to AR transmission from the environment.

## Introduction

There is a growing concern about antibiotic resistance in natural and clinical settings. The overuse, or misuse, of antibiotics in medicine and agricultural operations are major suspects for increased antibiotic resistance. Areas of elevated resistance are often found in the environment, which is most evident in locations where environmental pollution has affected the local abundance of resistance traits [1]–[3]. In fact, there are various known mechanisms by which resistance traits may be retained or propagated in the presence of elevated chemical stressors (e.g., quaternary ammonium compounds [4] and metals [1], [5], [6]), which can locally influence resistance markers in exposed microbial populations. However, less attention has been paid to the intrinsic capacity for natural environments to retain and promote resistance. Relationships between geochemical conditions, micro-organisms and human health have been long documented in terms of altered mobility and toxicity of metals (e.g., mercury and arsenic) [7], but the influence of background geochemical conditions on intrinsic ARG abundance has not been considered relative to AR proliferation. The practical question is whether elevated metal levels in soils increase the prevalence of antibiotic genes in the environment.

As background, some trace metals are essential at low concentrations for enzymatic biochemical processes in bacteria, albeit they also can be toxic at higher levels, causing damage to DNA and membranes [8], [9]. As an example, copper has been used as an antiseptic for many years [10] and, as such, metals like copper might act as environmental selectors that demand cell defence. Alternately, other metals like chromium and lead have no known function in bacterial cells, but can also cause oxidative stress [11]. In fact, bacterial resistance mechanisms exist to mitigate toxicity effects of excessive bio-available metals as part of their SOS (stress) response strategy [12]. Defence-associated metal resistance genes are often closely associated with those responsible for AR on mobile genetic elements. These genes can either encode for generic detoxifying mechanisms (e.g., efflux pumps), which non-specifically reduce intracellular concentrations of both metals and antibiotics (cross resistance), or may involve separate genes, which are integrated on the same genetic element (co-resistance).

In essence, the presence of one stressor is likely to select for the other; however, the extent to which soil-metal levels affect the selection of the resistant bacteria and AR gene levels is not known. To our knowledge, there is no experimental evidence or quantitative data that relate metal levels in the environment to ARG abundance, which we contend may be important to understanding the capacity of the natural environment to retain and transmit AR. As such, by elucidating relationships between metals and AR in theenvironment, we might better define how environmental reservoirs might influence AR transmission to clinically important strains. For example, genes can be harboured among environmental microorganisms, but with horizontal gene transfer, these traits can be shared with other bacteria, including pathogens, which was apparent for extended spectrum beta-lactamase genes found in soils prior to their appearance in clinical settings [13]. Specifically, the aim of this experiment is to determine whether such relationships exist between metal content and ARG in soils.

## Methods

**Figure 1:**
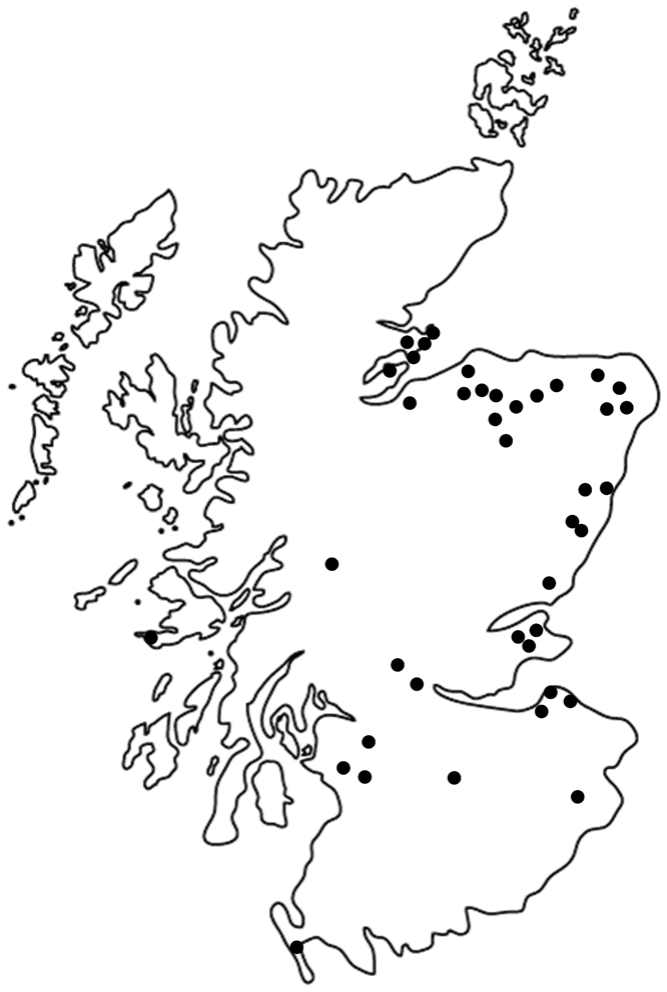
Distribution of sample locations in Scotland (n = 46).

We hypothesise that AR is directly related to geochemical conditions in the soils, and to test this hypothesis, we examined two sets of archived soils from The James Hutton Institute in Aberdeen in Scotland. Previous data had shown that DNA can be effectively obtained from dried archive soils [13], and the archive provided a collection of soils from throughout Scotland with good ancillary geochemical information. The first soil series comprised of 46 randomly selected archived soils originally collected across Scotland (see Fig. 1 and Supplemental Table S1), which were analysed for an array of ARG. This soil series was collected 1940 to early 1970s—very early in the antibiotic era, which provides a unique opportunity to view soils before human antibiotic use had likely much impact. The soil profiles sampled were each chosen to be typical of a soil series by the soil surveyor who mapped the soils in the area around the sampled profile. A single sample was generally taken around the mid-point of each soil horizon in the profile and we used the top sample from each profile for this study. While, there may be regional, non-point sources affecting sites (in a very broad sense; e.g., atmospheric deposition), we cannot attribute metal concentrations to any particular event or human activity in many locales. The soils were, in general, collected as part of a “national survey” to gain a representative sense of soil conditions.

As additional comparison, we also examined a second soil series comprised of soils collected in 2008 from experimental plots at Hartwood, North Lanarkshire and Auchincruive, South Ayrshire. These different plots had been provided sewage sludge amended with copper at 0, 50, 100 and 200 mg-copper/kg (between 1994 and 1998), respectively, and this series of impacted soils provided a valuable contrast to results from the first soil series. Details of the second set of soils can be found in Macdonald et al. [14], the experiment from which the archived soils were obtained. These soils were also archived at The James Hutton Institute.

### Soil collection and analysis

Archived samples were stored in climate-controlled rooms within The James Hutton Institute. According to archive records, soils were originally collected from multiple locations (0–25 cm) and pooled to obtain composite samples representative of each site. Soils are dried (30°C) and sieved for storage and analysis. Total-metal concentrations were determined by ICP (inductively coupled plasma) analysis, following *aqua regia* extraction [15]. These soils were extremely valuable and had limited availability, therefore we chose to use historical metal records, maintained at The James Hutton Institute, than re-analysing the samples with contemporary technology. Furthermore, the historic data is more representative of conditions under which the samples had been originally collected. Other analyses included soil pH, organic carbon/ash content, total phosphate, and particle size; again, information was based on archived records and were based on standard analytical methods [e.g., 15].

### DNA analysis

Long-term storage does not bias DNA results [16], [17]. Once retrieved from the archive, soils were sterilely weighed into prepared centrifuge tubes containing buffer and extraction beads (by weighing tubes before and after soil addition); usually 200–300 mg (as dry weight) of soil was used. Cells incubated in the buffer 15–20 minutes for rehydration. DNA was extracted from soil using FastDNA Spin Kit (MP Bio) according to manufacturer's instructions. DNA was eluted with 100-µL elution buffer and temporarily stored at -20°C; long-term storage was at −80°C.

### qPCR methods

Eleven determinants, targeting tetracycline resistance (*tet*), extended-spectrum beta-lactamases (*bla*), and erythromycin resistant methylases (*erm*), were chosen based on previous experience assessing metal-contaminated sediments [18] and their general ubiquity in the environment [16], [19]–[22]. Specifically, assays included *tet*(M), *tet*(Q) and *tet*(W) [19], which are three highly promiscuous (common) resistance traits that encode for ribosomal protection proteins against tetracyclines. *Tet*(B) [20], an efflux gene, was also tested but did not generate many positive results. Primers for *bla*_TEM_, *bla*_SHV_, *bla*_CTX-M_ and *bla*_OXA_ targeted conservative regions on the four common beta-lactamase genes (which encode for enzymes that inactivate penicillin and other beta-lactam antibiotics) [19], and specific primer pairs targeting *erm*(B), *erm*(C), *erm*(E) and *erm*(F) [22]. All ten determinants were quantified in the first experiment, whereas only *tet*(M), *tet*(W), *erm*(F), *bla*_TEM_, and *bla*_SHV_, and *bla*_CTX-M_ (based on current results and previous experience) were analysed in the second experiment.

Two microlitres of DNA template and appropriate primers were combined with iQ Supermix PCR reagent (BioRad, Hercules, CA) and molecular-grade water to create 25-µl volumes. Analyses were then performed using a BioRad iCycler equipped with iQ fluorescence detector and software (BioRad). Temperature cycles were 95°C (10 min), and then 40 cycles of 94°C (20 sec), annealing temperatures (*tet*- and *erm*- determinants: 60°C; *bla*_TEM_: 50°C, and 55°C for the remaining *bla* genes) for 60 seconds, and 72°C (for *bla*-genes only) for an additional 30 seconds. Samples were analysed in duplicate; any samples with a major discrepancy (high analytical variability, i.e., greater than one-cycle difference) were re-analysed. Typical duplicate values ranged within ±0.3 (*log* scale). SYBR-Green I, a non-specific fluorescent dye, was used and followed with a post-analytical temperature melt curve to verify reaction quality (50–95°C, ΔT = 0.1°C/second).

All reactions were run with serially diluted plasmid-DNA standards of known quantity, created from gene-positive bacteria. QPCR reaction efficiencies were determined by spiking sample with known amounts of DNA template; these results were compared with efficiencies of “neat” standards (plasmids dissolved in nanopure water). All samples were diluted, either 1∶100 or 1∶1000, to minimise inhibitory effects, often caused by substances from soils co-eluting with the DNA. Correlation coefficients (*r* ^2^) for all standard curves were >0.99; and *log* gene-abundance values (except those below detection limits) were within the linear range of the calibration curves. Any sample values below detection were not included in any statistical analyses.

### Data analysis

ARG abundances were normalised to 16S-rRNA gene abundances (a surrogate measure of ‘total bacteria’) to minimise variance caused by differential extraction and analytical efficiencies, and differences in background bacterial abundances. These normalised values are then *log*-transformed to normalise the data (Kolmogorov-Smirnov test).

All statistics were conducted using SPSS^TM^ version 18. Factors for analyses included: *log*-transformed abundances, which were determined *de novo* from retrieved archive samples. Other information, from archived records, included: “total metal” content of chromium, copper, molybdenum, nickel, and lead (Table S2); extractable measures of zinc and iron (no total values available); and soil quality characteristics—pH, organic carbon, total phosphate, sand, silt, clay and ash content (Table S1).

## Results and Discussion

The primary soil series was obtained from a wide range of soil conditions in Scotland. With these soils, we assessed the correlation of numerous metals and other soil parameters (e.g., organic carbon, total phosphorus, dry ash, pH, and silt, sand and clay contents) (Table 1), and the relative abundance of ARG in soils (Table S3). A bivariate correlation analysis (Table 2; other correlations can be found in Supplemental Table S4) showed that eight of the eleven quantified ARG positively correlated with soil copper levels; five ARG being highly significant (*p*<0.05), including *tet*(M), *tet*(W), *bla*_OXA_, *erm*(B) and *erm*(F) (all genes normalised to 16S-rRNA gene abundances). In addition, chromium positively correlated with relative *tet*(M), *bla*_CTX-M_ and *bla*_OXA_ gene abundances. Other metals also showed positive correlations, but were gene specific; e.g., nickel linked with *tet*(W), and *tet*(M) associated with nickel, lead and extractable iron, whereas only *erm*(B) had negative correlations with any metals (lead, zinc and iron). Overall, *tet*(M) level was most consistently linked to soil metal conditions, and copper most strongly influenced ARG abundances.

**Table 1:** Soil properties of samples collected throughout Scotland.

	Mean and 95% confidence intervals (in brackets)	Minimum – maximum
Cobalt (mg/kg)	10.2 (6.7)	0–140
Chromium (mg/kg)	53.1 (20.4)	0–250
Copper (mg/kg)	21.2 (9.2)	0–140
Nickel (mg/kg)	25.8 (7.4)	0–100
Lead (mg/kg)	52.4 (44.9)	10–1000
Zinc, extractable (meq/L)	2.5 (2.1)	0–38
Iron, extractable (meq/L)	23.0 (9.0)	0–115
Carbon, organic (%)	5.2 (2.1)	0–45.3
Phosphorus, total (mg/kg)	354 (145)	0–1610
pH	5.8 (0.2)	4.3–7.2
Sand (%)	45.3 (6.7)	0–88.0
Silt (%)	26.9 (4.5)	0–64.0
Clay (%)	14.3 (2.7)	0–45.6
Ash (%)	88.3 (3.7)	22–97

**Table 2:** Bi-variate correlations among geochemical properties and normalised gene abundances (*log*-transformed).

	*tet*(M)/16S	*tet*(Q)/16S	*tet*(W)/16S	*bla*_TEM_/16S	*bla*_SHV_/16S	*bla*_CTX_/16S	*bla*_OXA_/16S	*erm*(B)/16S	*erm*(C)/16S	*erm*(E)/16S	*erm*(F)/16S
Carbon, organic	−.123	.189	.227	−.080	.222	−.139	.094	−.764	.444*	−.058	.223
Phosphorus, total	.357	−.047	−.081	−.264	−.267	−.278	−.041	−.169	−.075	−.293	−.392 *
Sand	−.404	.145	.287	−.026	.447 **	.033	.064	−.366	.270	.183	.248
Silt	−.021	.238	−.138	−.140	−.151	.521 **	.172	.038	−.229	.415 **	.307
Clay	.377	.260	−.177	.042	−.398 *	.061	.000	.562	−.297	.153	−.005
Ash	−.076	.095	−.043	.017	.007	.308 *	−.021	.189	−.248	.322 *	.327
pH	.221	.251	−.154	−.059	.064	.602 **	.312 *	.923 *	−.053	.438 **	.208
Cobalt, total	.399	.179	.237	.167	.138	−.116	.137	−.115	−.173	−.085	.191
Chromium, total	.492 *	−.017	−.067	.045	−.039	.324 *	.390 **	−.101	.163	.053	.046
Copper, total	.439 *	.281	.296 *	.276	.280	.038	.408 **	.845 *	−.052	−.028	.432 *
Nickel, total	.336	.262	.363 *	.191	.148	−.121	.281	−.158	−.269	−.011	.066
Lead, total	.407	−.107	.105	.245	−.077	.047	.037	−.703	−.328	.110	−.014
Zinc, extractable	.157	−.333	.206	.192	.236	−.138	.215	−.583	.307	−.139	.211
Iron, extractable	.597 **	.241	.156	.060	.113	−.286	.124	−.621	.126	−.298	−.270

*Significant at *P*<0.05 level (α = 5%).

**Significant at *P*<0.01 level (α = 1%).

**Figure 2:**
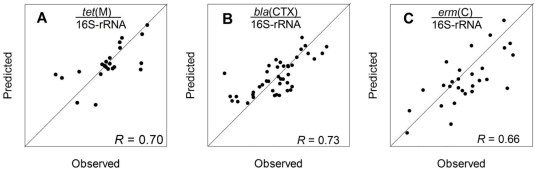
Comparison of multi-linear regression predictions and observed relative abundances of A) *tet*(M), B) *bla*_CTX-M_ and C) *erm*(F) genes. Factors entered into the regression included: total chromium, copper, nickel, lead, extractable iron, and pH.

Despite these significant individual correlations, we suspected that groups of factors were more likely to influence AR gene abundance in complex soil environments. Therefore, multiple-linear regression (MLR) also was performed on the data set (Fig. 2; Supplemental Table S5). Based on the MLR, stronger associations between soil conditions and ARG levels (i.e., *R*>0.70) become apparent, although these correlations are consistent with bivariate correlation predictions. As examples, observed *tet*(M) (Figure 2A; *R* = 0.70, *p* = 0.11), *bla*_CTX-M_ (Figure 2B; *R* = 0.73, *p*<0.01) and *erm*(F) (Figure 2C; *R* = 0.66, *p* = 0.05) abundances correlated to a model based on five metals (chromium, copper, nickel, lead and iron) and pH, and explained ∼50% of the variability in gene abundances. Other MLR relationships between metals and relative gene abundances were also found, but observations were not as strongly linked to predictions (See Supplemental Fig. S1).

Regardless of statistical approach, soil copper appears to be a key major factor in soil ARG (as well as chromium and iron; Supplemental Table S5) with 2–3 orders of magnitude of differences in intrinsic ARG levels attributable to five metals and pH. It should be noted our coefficients of determination (*R^2^*) for *tet*(M), *bla*_CTX-M_, and *erm*(F) suggest only about 50% of the variance is explained by the model. However, this correlation is impressive given the complexity of soil environments and the fact it is difficult to parameterise all possible factors that might contribute to AR. Given the age and limited information on most of the soils, this correlation is surprisingly strong and suggests that similar key factors may contribute to intrinsic ARG levels.

**Figure 3:**
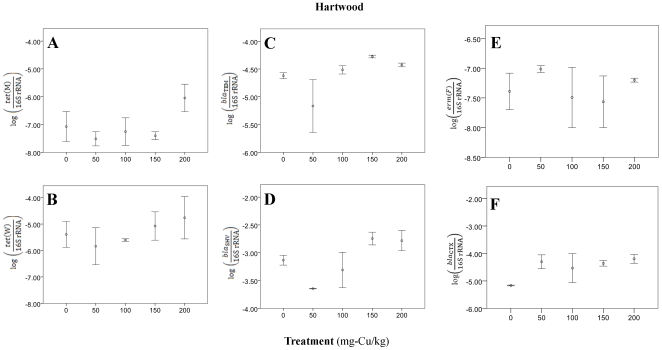
Relative resistant gene levels in experimental plots, located at Hartford, North Lanarkshire, receiving copper-amended sewage sludge: 0, 50, 100 or 200 mg-copper/kg.

**Figure 4:**
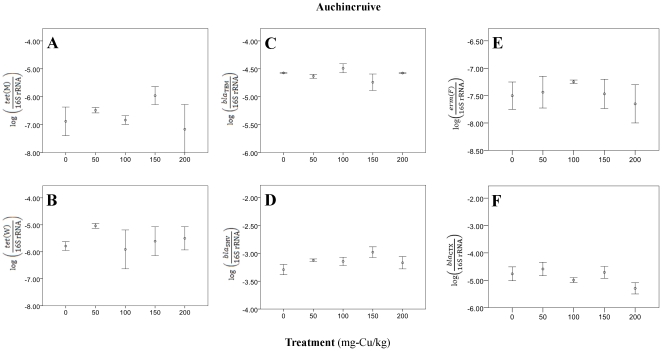
Relative resistant gene levels in experimental plots, located at Auchincruive, South Ayrshire, receiving copper-amended sewage sludge: 0, 50, 100 or 200 mg-copper/kg.

To assess how ARG patterns in the soils from across Scotland compared with more metal-impacted soils (e.g., with Cu), soils from experimental plots that had been provided Cu-amended sewage sludge at different levels and sites were quantified for ARG. Significant treatment differences were found at the Hartwood site (North Lanarkshire; Fig. 3; Supplemental Table S6), which had relatively higher *bla*_CTX-M_ relative gene abundances (*ANOVA*; *F*_4,6_ = 3.17, *p* = 0.10) when Cu levels were supplemented. Further, *bla*_TEM_ and *bla*_SHV_ gene abundances were elevated when Cu levels were greater than 150 mg/kg at Hartwood (*ANOVA*; *bla*_TEM_: *F*_4,7_ = 3.41, *p* = 0.07; *bla*_SHV_: *F*_4,7_ = 4.54, *p* = 0.04). *Tet*(M) and *tet*(W) also showed general upward trends at this site, but were not significant (*ANOVA*; all *p*>0.10). The second site (Auchincruive, South Ayrshire; Fig. 4; Supplemental Table S6) did not show any significant evidence of elevated resistance potential with increased Cu-content for many genes (*ANOVA*; *p*>0.17).

We cannot rule out that bacteria with resistance traits in the original sludge or residual antibiotics in the sludge might have been sources of the ARG. However, these samples were collected in 2008, and the elevated Cu content in the treatments was still apparent from soil analysis ten years after Cu amendments. Overall, Cu-amended plots had slightly higher ARG than plots without Cu amendment, although metal effects were less well defined than in the broader soil archive. Also, MLR equations from the first soil series poorly predicted ARG levels in the impacted soils, which imply artificial metal additions may alter intrinsic ARG and metal patterns, and may not accurately reflect native ARG-metal relationships in soils.

Regardless of specific correlations, both experiments show that soil conditions can affect the intrinsic potential for AR in associated microbial communities, which is noteworthy because it is the first demonstration that relatively low total metal levels correlate with ARG abundance in soils. Even more interestingly, our results suggest that even low metal levels may co-select for antibiotic resistance. In reality, metal impacts on AR signatures at environmental sites are not new; however, all previous work has been done at heavily polluted sites. For example, Stepanauskas et al. [2] found higher levels of AR in the water leaving a settling basin, which contained ash from coal-fired power stations known to have elevated metals. Further to this, Wright et al. [21] looked at how AR in a metal polluted river compared to that of a pristine reference stream. They found metal and antibiotic resistance to be highest in the sediment bacteria. Similar results were found by Graham et al. [18]; elevated levels of resistance were found surrounding discharges of metal-laden leachate from landfill in Cuba. Noteworthy, Berg et al. [5] linked increased soil copper exposure to increased levels of antibiotic resistance. In all aforementioned cases, sediment acted as a sink for metals and promoted higher concentrations of AR-related genes. In another experiment taking advantage of defined copper sulphate additions to soils [6], researchers found significantly higher ampicillin resistance in susceptibility tests among copper-resistant bacteria. AR patterns can be related to total metals and also to bioavailable copper levels [22]; however, it should be noted total metal concentrations are often poor predictors of bioavailability and toxicity; many factors in soil are attributed to this [23].

However, here we show that specific ARG concentrations themselves significantly correlate with metal conditions, but relationships between metal conditions in natural soils differ than impacted soils, which is critical for assessing broader AR risk from soil sources. Clearly, elevated AR can be found in polluted areas, but Figure 2 shows that lower metal concentrations influence intrinsic AR potential and that influence differs from impacted soils. Therefore, investigations such as ours are important to better understand the risks of AR, mechanisms that promote AR propagation, and background source inputs to AR in anthropogenically important species.

Environmental bacteria can harbour resistance genes, and the constant exposure to metals can increase their frequency in the gene pool [24]. With gene transfer occurring among soil bacteria, the chance of acquiring resistant pathogens, therefore, is increased. Epidemiological studies are now suggested to further examine whether intrinsic resistance actually does contribute to increased AR, especially at landscape and larger scales. As such, this new information from soils becomes crucial for developing management strategies to reduce the global risk of AR and protecting agricultural and aqua-cultural economies by better knowing where AR might germinate.

## Supporting Information

Figure S1 Comparison of multiple linear regression predictions and observed relative abundances of genes.
(PDF)

Table S1 Original location and physical-chemical properties of archived samples.
(DOCX)

Table S2 Metal concentrations of archive soil used in the study.
(DOCX)

Table S3 Distribution of physical and chemical conditions.
(DOCX)

Table S4 Bi-variate correlations among physical-chemical properties.
(DOCX)

Table S5 Results of the multi-linear regression analyses.
(DOCX)

Table S6 One-way *ANOVA* statistics describing treatment differences in Hartwood and Auchincruive Cu-amended agricultural plots.
(DOCX)
