# Supplementary figures and images for: Antibiotic Resistance Gene Abundances Correlate with Metal and Geochemical Conditions in Archived Scottish Soils
Source: PLoS One. 2011 Nov 9;6(11):e27300. doi: 10.1371/journal.pone.0027300 (PMC3212566; doi:10.1371/journal.pone.0027300)

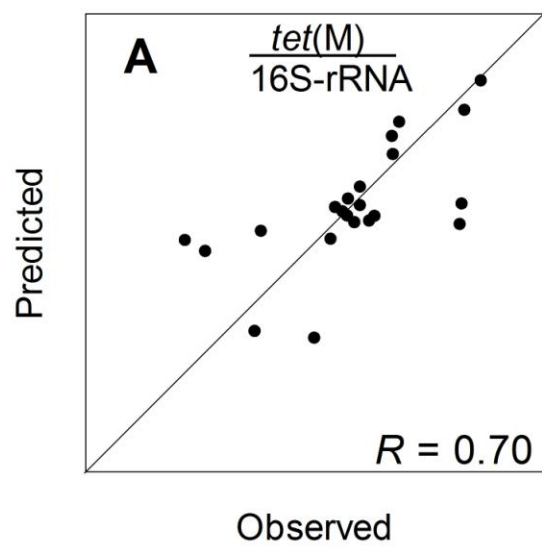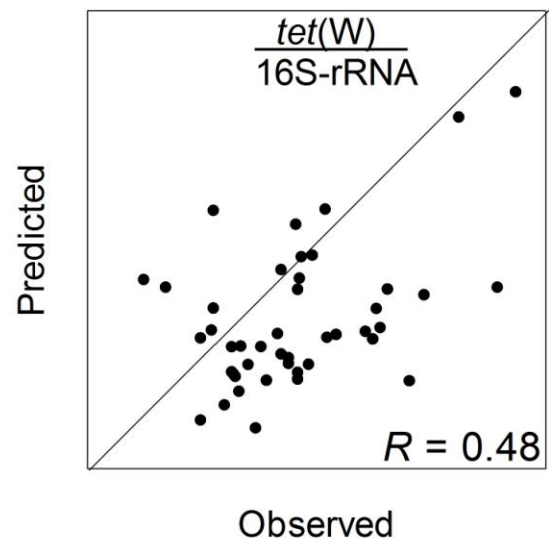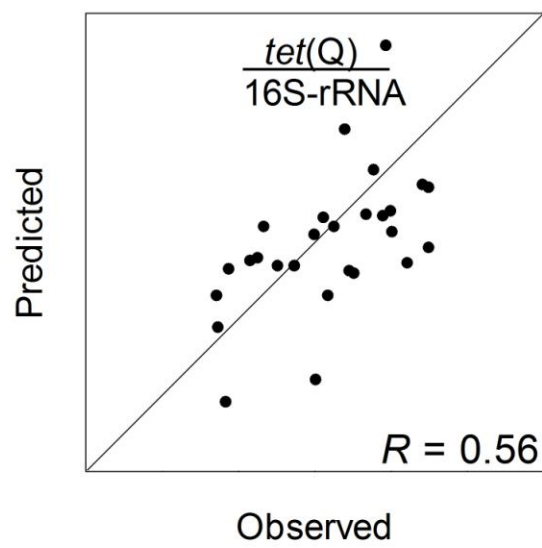

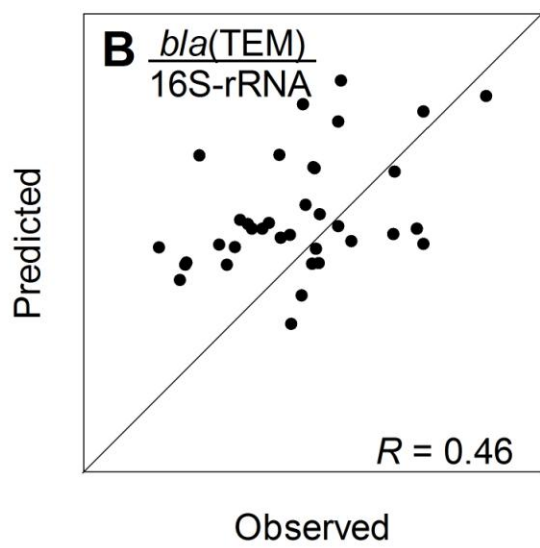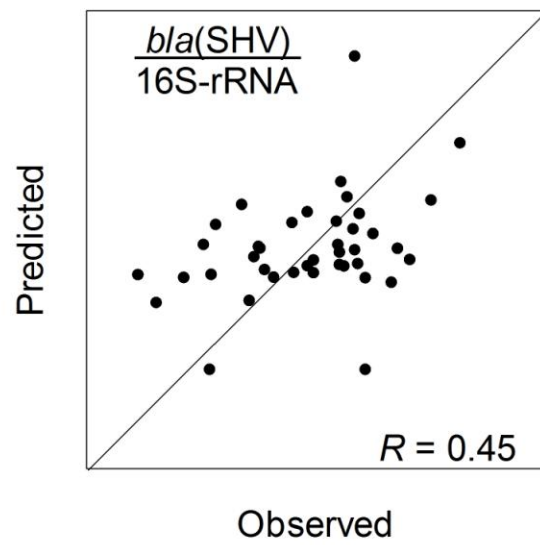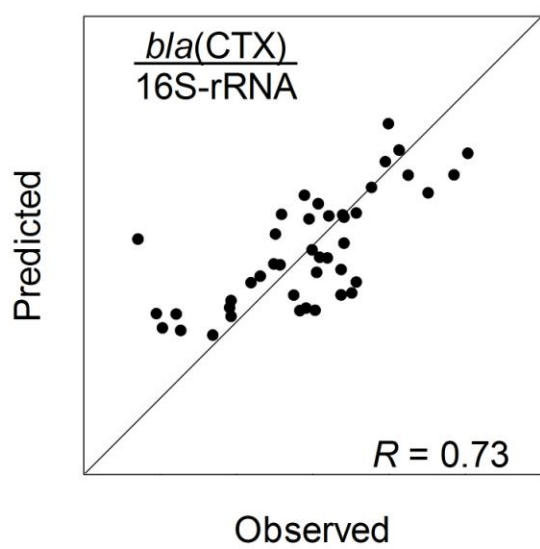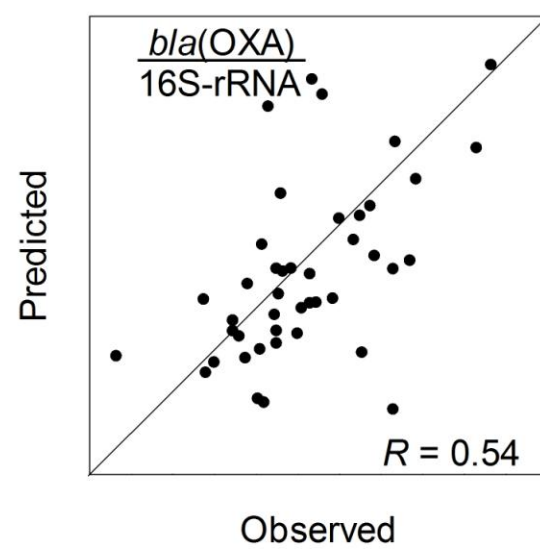

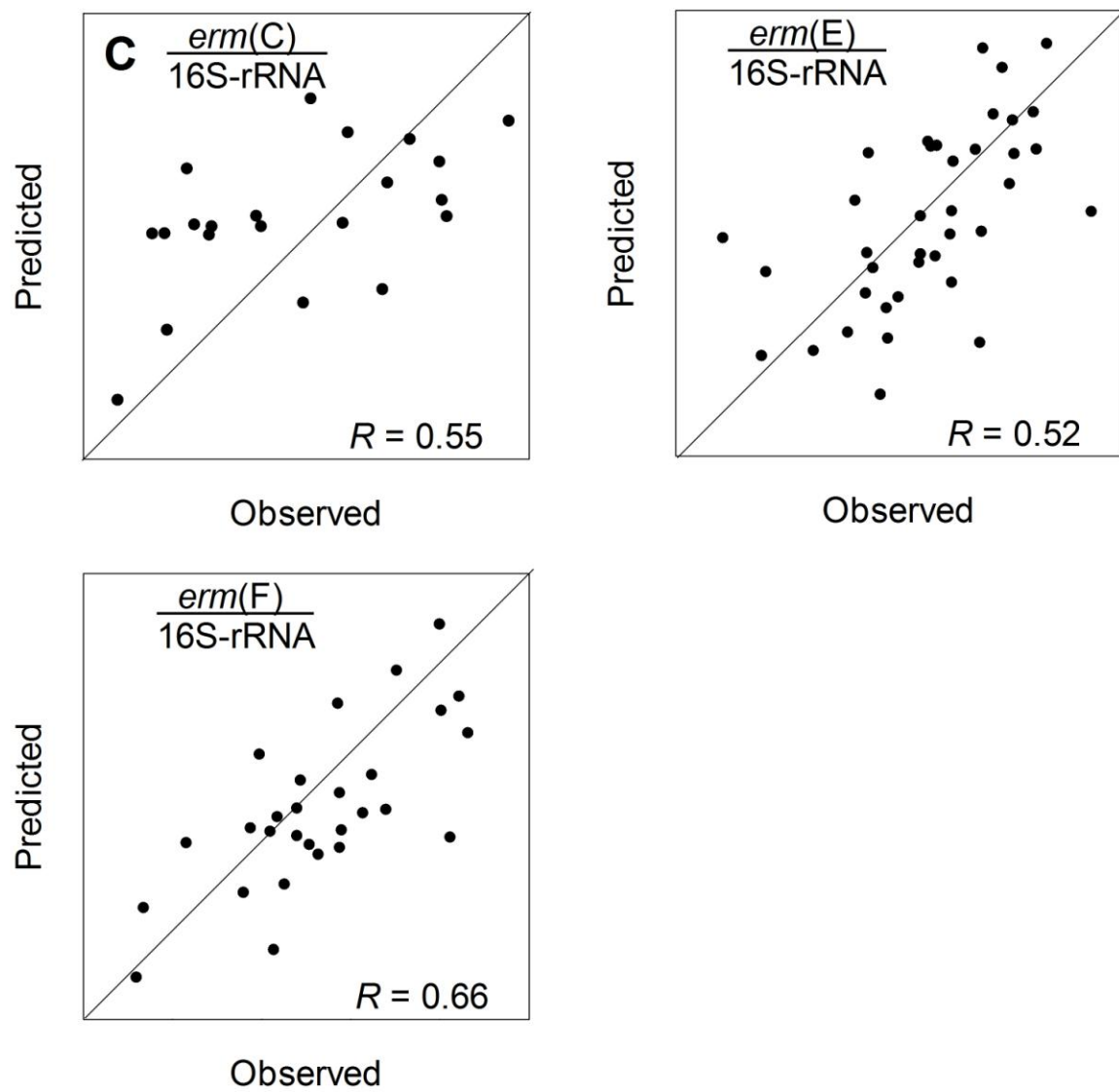

Figure 2

Supplement: Figure S1 — Comparison of multiple linear regression predictions and observed relative abundances of genes. (PDF) [file pone.0027300.s001.pdf]
