# Supplementary material for: Antibiotic Resistance Gene Abundances Correlate with Metal and Geochemical Conditions in Archived Scottish Soils
Source: PLoS One. 2011 Nov 9;6(11):e27300. doi: 10.1371/journal.pone.0027300 (PMC3212566; doi:10.1371/journal.pone.0027300)
Supplement: Table S1 — Original location and physical-chemical properties of archived samples. (DOCX) [file pone.0027300.s002.docx]

**Supplemental Table S1**. Original location and physical-chemical character of archived samples.

|  |  | **Location^a^** | |  |  |  |  |  |  |  |  |
| --- | --- | --- | --- | --- | --- | --- | --- | --- | --- | --- | --- |
| **Sample** | **Year** | **Easting** | **Northing** | **Characteristics** | **pH** | **Organic**  **Carbon** | **Total**  **phosphate** | **Sand** | **Silt** | **Clay** | **Ash** |
| 54146 | 1946 | Unknown | unknown | Unknown | 5.5 | 20.3 | 1280 | 45.7 | 8.4 | 15.8 | 65 |
| 54147 | 1946 |  |  | Unknown | 5.3 | 11.1 | 755 | 68.6 | 12.5 | 10.7 | 83 |
| 61616 | 1947 | 383200 | 783200 | Humus-iron podzol | 5.2 | 7.2 | 1160 | 55.3 | 19.8 | 17.0 | 85 |
| 79395 | 1949 | 356200 | 854900 | Non-calcareous gley | 5.8 | 2.2 | 695 | 0.0 | 0.0 | 17.8 | 93 |
| 82036 | 1950 | 245100 | 641000 | Non-calcareous gley | 5.5 | 4.3 | 1330 | 0.0 | 0.0 | 39.9 | 84 |
| 82203 | 1950 | 227300 | 646200 | Humic gley | 6.0 | 0.0 | 1410 | 0.0 | 0.0 | 0.0 | 65 |
| 111073 | 1956 | 365500 | 626400 | Brown forest soil | 5.4 | 7.5 | 885 | 45.9 | 24.7 | 13.9 | 79 |
| 114415 | 1956 | 365700 | 769900 | Brown forest soil with gleying | 6.7 | 1.6 | 0 | 35.6 | 36.5 | 25.4 | 96 |
| 114420 | 1956 | 365800 | 770000 | Non-calcareous gley | 6.6 | 1.6 | 0 | 51.2 | 28.0 | 18.5 | 95 |
| 119387 | 1957 | 385400 | 842100 | Humus-iron podzol | 6.1 | 6.0 | 1610 | 33.1 | 49.0 | 11.5 | 87 |
| 126831 | 1958 | 387200 | 787300 | Brown forest soil with gleying | 6.2 | 4.7 | 253 | 28.2 | 33.0 | 32.7 | 90 |
| 136234 | 1959 | 344300 | 673700 | Brown forest soil with gleying | 5.9 | 1.5 | 0 | 57.0 | 22.2 | 17.6 | 95 |
| 152064 | 1961 | 329300 | 732300 | Brown forest soil | 6.5 | 3.9 | 0 | 37.2 | 39.6 | 18.0 | 90 |
| 152083 | 1961 | 349200 | 735300 | Brown forest soil | 6.2 | 4.5 | 0 | 39.9 | 39.7 | 14.4 | 88 |
| 152084 | 1961 | 349200 | 735300 | Brown forest soil | 6.2 | 3.7 | 0 | 36.8 | 43.1 | 14.9 | 90 |
| 155615 | 1961 | 281700 | 874000 | Humus-iron podzol | 5.8 | 2.0 | 0 | 61.0 | 24.0 | 13.0 | 96 |
| 155616 | 1961 | 281700 | 874000 | Humus-iron podzol | 5.1 | 1.6 | 0 | 53.0 | 31.0 | 16.0 | 97 |
| 155684 | 1961 | 271100 | 869900 | Humus-iron podzol | 6.2 | 5.3 | 0 | 52.0 | 30.0 | 14.0 | 92 |
| 155756 | 1961 | 359100 | 679400 | Non-calcareous gley | 6.8 | 3.9 | 821 | 3.8 | 43.9 | 45.6 | 87 |
| 164260 | 1962 | 271100 | 870600 | Immature raised beach soil | 5.4 | 5.5 | 0 | 63.0 | 27.0 | 5.0 | 89 |
| 164261 | 1962 | 271100 | 870600 | Immature raised beach soil | 5.4 | 5.3 | 0 | 63.0 | 24.0 | 8.0 | 90 |
| 164320 | 1962 | 286600 | 879400 | Humus-iron podzol | 5.6 | 2.5 | 0 | 60.0 | 26.0 | 11.0 | 95 |
| 164338 | 1962 | 287300 | 879400 | Not applicable | 5.8 | 2.4 | 0 | 65.0 | 20.0 | 12.0 | 95 |
| 165456 | 1962 | 348400 | 679900 | Brown forest soil | 6.0 | 1.9 | 0 | 69.8 | 14.3 | 13.2 | 96 |
| 189123 | 1965 | 276800 | 693200 | Non-calcareous gley | 5.6 | 3.6 | 670 | 13.1 | 53.4 | 29.0 | 92 |
| 191195 | 1965 | 266602 | 702800 | Brown forest soil with gleying | 5.8 | 3.9 | 883 | 42.3 | 34.9 | 17.4 | 91 |
| 194665 | 1964 | 285600 | 855900 | Humus-iron podzol | 5.6 | 1.6 | 0 | 88.0 | 7.0 | 5.0 | 97 |
| 205659 | 1966 | 386600 | 837600 | Humus-iron podzol | 5.3 | 2.6 | 1300 | 55.1 | 20.2 | 20.0 | 91 |
| 205664 | 1966 | 387100 | 837200 | Iron podzol | 5.8 | 9.8 | 840 | 39.8 | 33.1 | 11.0 | 79 |
| 207571 | 1967 | 341500 | 849900 | Non-calcareous gley | 4.8 | 2.8 | 136 | 58.5 | 23.8 | 14.0 | 93 |
| 208386 | 1966 | 333700 | 712900 | Brown forest soil with gleying | 6.1 | 3.9 | 0 | 63.2 | 19.1 | 13.4 | 91 |
| 209070 | 1967 | 207400 | 547600 | Brown forest soil | 6.0 | 5.2 | 0 | 69.0 | 12.0 | 13.0 | 89 |
| 241845 | 1971 | 276700 | 843800 | Humus-iron podzol | 5.1 | 3.7 | 0 | 56.0 | 32.0 | 8.0 | 93 |
| 242310 | 1971 | 236500 | 728700 | Not applicable | 4.3 | 45.3 | 0 | 0.0 | 0.0 | 0.0 | 22 |
| 243105 | 1971 | 294600 | 850900 | Humus-iron podzol | 5.8 | 1.9 | 0 | 60.0 | 30.0 | 10.0 | 97 |
| 245014 | 1971 | 325300 | 841800 | Humus-iron podzol | 5.7 | 2.4 | 311 | 65.0 | 22.6 | 9.2 | 94 |
| 245052 | 1971 | 326200 | 839800 | Humus-iron podzol | 7.2 | 6.0 | 442 | 34.4 | 52.2 | 6.4 | 87 |
| 250136 | 1971 | 300400 | 634300 | Alluvial soil (silty) | 6.1 | 1.8 | 0 | 22.0 | 64.0 | 12.0 | 95 |
| 253915 | 1972 | 172300 | 731400 | Brown forest soil | 5.2 | 9.2 | 0 | 30.6 | 43.4 | 10.8 | 82 |
| 254187 | 1972 | 317100 | 842800 | Non-calcareous gley | 6.3 | 3.7 | 104 | 36.0 | 52.0 | 9.0 | 92 |
| 269249 | 1973 | 309500 | 858800 | Humus-iron podzol | 5.8 | 2.2 | 144 | 64.0 | 28.0 | 8.0 | 95 |
| 269264 | 1973 | 318500 | 830900 | Humus-iron podzol | 6.9 | 3.9 | 216 | 67.0 | 25.0 | 8.0 | 92 |
| 279796 | 1974 | 318900 | 839600 | Non-calcareous gley | 6.3 | 3.4 | 173 | 80.0 | 15.0 | 5.0 | 93 |
| 279820 | 1974 | 329100 | 846500 | Alluvial soil (sandy) | 6.1 | 1.8 | 163 | 67.0 | 17.0 | 16.0 | 97 |

^a^ Geographic Cartesian coordinates based on the UK Ordnance Survey.
