# Supplementary material for: Antibiotic Resistance Gene Abundances Correlate with Metal and Geochemical Conditions in Archived Scottish Soils
Source: PLoS One. 2011 Nov 9;6(11):e27300. doi: 10.1371/journal.pone.0027300 (PMC3212566; doi:10.1371/journal.pone.0027300)
Supplement: Table S2 — Metal concentrations of archive soil used in the study. (DOCX) [file pone.0027300.s003.docx]

**Supplemental Table S2**. Metal concentrations of archive soils used in the study.

| **Sample** | **Total**  **Cobalt** | **Total Chromium** | **Total Copper** | **Total**  **Nickel** | **Total**  **Lead** | **Extractable Zinc** | **Extractable Iron** |
| --- | --- | --- | --- | --- | --- | --- | --- |
| 54146 | 140 | 65 | 140 | 100 | 35 | 0.0 | 55 |
| 54147 | 40 | 100 | 100 | 80 | 20 | 0.0 | 40 |
| 61616 | 20 | 200 | 50 | 70 | 200 | 0.0 | 115 |
| 79395 | 0 | 0 | 0 | 0 | 0 | 0.0 | 35 |
| 82036 | 0 | 0 | 0 | 0 | 0 | 0.0 | 60 |
| 82203 | 10 | 100 | 15 | 25 | 150 | 0.0 | 60 |
| 114415 | 15 | 20 | 40 | 40 | 15 | 0.0 | 20 |
| 114420 | 0 | 0 | 30 | 0 | 0 | 0.0 | 0 |
| 119387 | 0 | 0 | 20 | 0 | 0 | 0.0 | 0 |
| 126831 | 7 | 70 | 6 | 30 | 10 | 0.0 | 0 |
| 136234 | 20 | 200 | 40 | 60 | 90 | 5.0 | 58 |
| 152064 | 10 | 30 | 30 | 30 | 100 | 0.0 | 21 |
| 152083 | 25 | 250 | 60 | 40 | 18 | 0.0 | 20 |
| 152084 | 20 | 200 | 80 | 40 | 20 | 0.0 | 34 |
| 155615 | 20 | 200 | 100 | 40 | 20 | 0.0 | 36 |
| 155616 | bdl | 0 | 2 | 15 | 6 | 0.4 | 30 |
| 155684 | bdl | 0 | 2 | 20 | 10 | 0.4 | 24 |
| 155756 | bdl | 0 | 3 | 15 | 10 | 0.0 | 0 |
| 164260 | 0 | 100 | 8 | 25 | 50 | 0.0 | 0 |
| 164261 | bdl | 0 | 2 | 20 | 8 | 6.6 | 51 |
| 164320 | 3 | 0 | 3 | 30 | 4 | 0.0 | 0 |
| 164338 | bdl | 0 | 3 | 20 | 15 | 0.0 | 24 |
| 165456 | bdl | 0 | 2 | 15 | 10 | 0.7 | 68 |
| 189123 | 10 | 30 | 20 | 40 | 60 | 0.0 | 27 |
| 191195 | 15 | 150 | 10 | 40 | 40 | 0.0 | 42 |
| 194665 | 10 | 0 | 10 | 40 | 30 | 0.0 | 49 |
| 205659 | bdl | 10 | 2 | -10 | 50 | 0.5 | 31 |
| 205664 | 15 | 100 | 20 | 30 | 15 | 0.0 | 15 |
| 207571 | 6 | 50 | 2 | 20 | 15 | 0.0 | 59 |
| 208386 | 4 | 40 | 8 | 15 | 30 | 38 | 7 |
| 209070 | 6 | 60 | 4 | 20 | 25 | 10 | bdl |
| 241845 | 20 | 0 | 30 | 80 | 100 | 17 | 13 |
| 243105 | bdl | 10 | 4 | 0 | 50 | 0.0 | 13 |
| 245014 | 0 | 0 | 0 | 0 | 0 | 25 | 36 |
| 245052 | bdl | 10 | 3 | 10 | 20 | 0.0 | 16 |
| 250136 | 0 | 0 | 0 | 0 | 0 | 0.0 | bdl |
| 253915 | 6 | 60 | 20 | 15 | 15 | 0.0 | 27 |
| 254187 | 15 | 0 | 25 | 60 | 1,000 | 1.0 | 17 |
| 269249 | 15 | 100 | 25 | 40 | 8 | 0.0 | bdl |
| 269264 | 0 | 0 | 0 | 0 | 0 | 0.0 | bdl |
| 279796 | 3 | 20 | 3 | 4 | 10 | 0.0 | bdl |
| 279820 | 3 | 40 | 3 | 10 | 15 | 0.0 | bdl |
