# Supplementary material for: Antibiotic Resistance Gene Abundances Correlate with Metal and Geochemical Conditions in Archived Scottish Soils
Source: PLoS One. 2011 Nov 9;6(11):e27300. doi: 10.1371/journal.pone.0027300 (PMC3212566; doi:10.1371/journal.pone.0027300)
Supplement: Table S3 — Distribution of physical and chemical conditions. (DOCX) [file pone.0027300.s004.docx]

**Supplemental Table S3**. Distribution of physical and chemical conditions among sampled soils.

|  | **Minimum** | **25% (Q1)** | **50% (median)** | **75% (Q3)** | **Maximum** |
| --- | --- | --- | --- | --- | --- |
| $\log\left[ \frac{tet\left( M \right)}{16SrRNA} \right]$ | -4.91 | -3.31 | -3.03 | -2.64 | -1.68 |
| $\log\left[ \frac{tet\left( Q \right)}{16SrRNA} \right]$ | -5.14 | -4.79 | -4.37 | -4.04 |  |
| $\log\left[ \frac{tet\left( W \right)}{16SrRNA} \right]$ | -3.65 | -3.19 | -2.90 | -2.64 | -1.66 |
| $\log\left[ \frac{{bla}_{\mathrm{TEM}}}{16SrRNA} \right]$ | -5.96 | -5.25 | -4.83 | -4.55 | -3.42 |
| $\log\left[ \frac{{bla}_{\mathrm{SHV}}}{16SrRNA} \right]$ | -5.16 | -4.38 | -3.94 | -3.73 | -3.05 |
| $\log\left[ \frac{{bla}_{\mathrm{CTX}-M}}{16SrRNA} \right]$ | -3.64 | -2.84 | -2.48 | -2.29 | -1.48 |
| $\log\left[ \frac{{bla}_{\mathrm{OXA}}}{16SrRNA} \right]$ | -4.07 | -3.38 | -3.23 | -2.90 | -2.27 |
| $\log\left[ \frac{erm\left( C \right)}{16SrRNA} \right]$ | -5.06 | -4.75 | -4.31 | -3.97 | -3.48 |
| $\log\left[ \frac{erm\left( E \right)}{16SrRNA} \right]$ | -5.02 | -3.70 | -3.28 | -2.93 | -2.20 |
| $\log\left[ \frac{erm\left( F \right)}{16SrRNA} \right]$ | -5.20 | -4.44 | -4.21 | -3.86 | -3.34 |
| Carbon, organic | 0.0 | 2.5 | 3.7 | 5.3 | 45.3 |
| Phosphorus, total | 0.0 | 0.0 | 52.0 | 710 | 1610 |
| Sand | 0.0 | 35.3 | 52.5 | 63.1 | 88.0 |
| Silt | 0.0 | 18.6 | 25.5 | 35.3 | 64.0 |
| Clay | 0.0 | 9.2 | 13.1 | 17.1 | 45.6 |
| Ash | 22.0 | 87.0 | 91.5 | 95.0 | 97.0 |
| pH | 4.3 | 5.5 | 5.8 | 6.2 | 7.2 |
| Cobalt, total | 0.0 | 0.0 | 5.0 | 15.0 | 140 |
| Chromium, total | 0.0 | 0.0 | 25.0 | 85.0 | 250 |
| Copper, total | 0.0 | 2.8 | 7.0 | 26.3 | 140 |
| Nickel, total | 0.0 | 9.5 | 20.0 | 40.0 | 100 |
| Lead, total | 0.0 | 9.5 | 15.0 | 36.3 | 1000 |
| Zinc, extractable | 0.0 | 0.0 | 0.0 | 0.7 | 38.0 |
| Iron, extractable | 0.0 | 0.0 | 20.5 | 37.0 | 115 |
