# Supplementary material for: Antibiotic Resistance Gene Abundances Correlate with Metal and Geochemical Conditions in Archived Scottish Soils
Source: PLoS One. 2011 Nov 9;6(11):e27300. doi: 10.1371/journal.pone.0027300 (PMC3212566; doi:10.1371/journal.pone.0027300)
Supplement: Table S4 — Bi-variate correlations among physical-chemical properties. (DOCX) [file pone.0027300.s005.docx]

**Supplemental Table S4**. Bi-variate correlations among physical-chemical properties of archived soil samples.

|  | Total cobalt | | Total chromium | | Total copper | | Total nickel | | Total Lead | | Extract. zinc | | Extract. iron | |  |  |
| --- | --- | --- | --- | --- | --- | --- | --- | --- | --- | --- | --- | --- | --- | --- | --- | --- |
| Sand | | -.053 | | -.205 | | -.018 | | -.006 | | -.167 | | -.008 | | -.128 | | |
| Silt | | -.099 | | .241 | | .022 | | .079 | | .311** | | -.212 | | -.304** | | |
| Clay | | .107 | | .262* | | .102 | | .151 | | -.025 | | -.150 | | .101 | | |
| Ash | | -.305** | | -.072 | | -.194 | | -.149 | | .057 | | -.354** | | -.206 | | |
| pH | | -.063 | | .167 | | .033 | | -.076 | | .054 | | -.409*** | | -.165 | | |
| Phosphorus,  total | | .371** | | .208 | | .183 | | .290* | | -.035 | | -.208 | | .387*** | | |
| Carbon, organic | | .324** | | -.007 | | .211 | | .157 | | -.099 | | .398*** | | .119 | | |
| Cobalt, total | | - | | .333** | | .821*** | | .741*** | | .080 | | -.067 | | .207 | | |
| Chromium,  total | | .333** | | - | | .565*** | | .463*** | | -.011 | | -.120 | | .225 | | |
| Copper, total | | .821*** | | .565*** | | - | | .737*** | | .071 | | -.124 | | .240 | | |
| Nickel, total | | .741*** | | .463*** | | .737*** | | - | | .321** | | -.018 | | .279* | | |
| Lead, total | | .080 | | -.011 | | .071 | | .321** | | - | | -.008 | | .099 | | |
| Zinc,extractable | | -.067 | | -.120 | | -.124 | | -.018 | | -.008 | | - | | -.046 | | |
| Iron, extractable | | .207 | | .225 | | .240 | | .279* | | .099 | | -.046 | | - | |  |

| ***** | Significant at *P* < 0.100 level (α = 10%). |
| --- | --- |
| ****** | Significant at *P* < 0.050 level (α = 5%). |
| ******* | Significant at *P* < 0.010 level (α = 1%). |
