# Supplementary material for: Antibiotic Resistance Gene Abundances Correlate with Metal and Geochemical Conditions in Archived Scottish Soils
Source: PLoS One. 2011 Nov 9;6(11):e27300. doi: 10.1371/journal.pone.0027300 (PMC3212566; doi:10.1371/journal.pone.0027300)
Supplement: Table S5 — Results of the multi-linear regression analyses. (DOCX) [file pone.0027300.s006.docx]

**Supplemental Table S5**. Results of multi-linear regression.

$$log\left[ \frac{tet(M)}{16SrRNA} \right]$$

| ***R*** = | .695 | ***R^2^*** = | .480 | ***P*** *=* | 0.112 |
| --- | --- | --- | --- | --- | --- |
|  |  |  |  |  |  |
|  | **Coefficient (B)** | **Standard**  **error** | **Standardised Coefficients (*β*)** | ***t*-score** | **Signific.**  **(*p*)** |
| Constant | -4.54 | 2.13 |  | -2.13 | .051 |
| Chromium, total | .0018 | .0056 | .163 | .318 | .755 |
| Copper, total | .0053 | .0106 | .169 | .494 | .629 |
| Nickel, total | -.0012 | .0173 | -.026 | -.069 | .946 |
| Lead, total | .0054 | .0105 | .136 | .515 | .615 |
| Iron, extractable | .0128 | .0067 | .427 | 1.91 | .077 |
| pH | .175 | .348 | .105 | .503 | .622 |

$$log\left[ \frac{tet(Q)}{16SrRNA} \right]$$

| ***R*** = | .555 | ***R^2^*** = | .308 | ***P*** *=* | .234 |
| --- | --- | --- | --- | --- | --- |
|  |  |  |  |  |  |
|  | **Coefficient (B)** | **Standard**  **error** | **Standardised Coefficients (*β*)** | ***t*-score** | **Signific.**  **(*p*)** |
| Constant | -6.61 | 1.16 |  | -5.71 | .000 |
| Chromium, total | -.0024 | .0016 | -.367 | -1.45 | .164 |
| Copper, total | .0026 | .0046 | .199 | .558 | .583 |
| Nickel, total | .0069 | .0066 | .345 | 1.04 | .309 |
| Lead, total | -.0022 | .0028 | -.168 | -.798 | .434 |
| Iron, extractable | .0023 | .0030 | .160 | .763 | .454 |
| pH | .356 | .190 | .395 | 1.87 | .076 |

$$log\left[ \frac{tet(W)}{16SrRNA} \right]$$

| ***R*** = | .482 | ***R^2^*** = | .232 | ***P*** *=* | .124 |
| --- | --- | --- | --- | --- | --- |
|  |  |  |  |  |  |
|  | **Coefficient (B)** | **Standard**  **error** | **Standardised Coefficients (*β*)** | ***t*-score** | **Signific.**  **(*p*)** |
| Constant | -2.81 | .746 |  | -3.77 | .001 |
| Chromium, total | -.0022 | .0011 | -.367 | -2.00 | .053 |
| Copper, total | .0031 | .0032 | .236 | .988 | .330 |
| Nickel, total | .0056 | .0041 | .336 | 1.36 | .181 |
| Lead, total | -.0001 | .0004 | -.031 | -.190 | .850 |
| Iron, extractable | .0011 | .0021 | .082 | .528 | .601 |
| pH | -.029 | .124 | -.037 | -.235 | .816 |

$$log\left[ \frac{{bla}_{\mathrm{TEM}}}{16SrRNA} \right]$$

| ***R*** = | .456 | ***R^2^*** = | .208 | ***P*** *=* | .344 |
| --- | --- | --- | --- | --- | --- |
|  |  |  |  |  |  |
|  | **Coefficient (B)** | **Standard**  **error** | **Standardised Coefficients (*β*)** | ***t*-score** | **Signific.**  **(*p*)** |
| Constant | -4.94 | 1.16 |  | -4.26 | .000 |
| Chromium, total | -.0033 | .0021 | -.398 | -1.58 | .125 |
| Copper, total | .0132 | .0064 | .593 | 2.06 | .049 |
| Nickel, total | -.0045 | .0067 | -.183 | -.674 | .506 |
| Lead, total | .0057 | .0032 | .436 | 1.80 | .084 |
| Iron, extractable | -.0026 | .0036 | -.149 | -.729 | .472 |
| pH | .001 | .195 | .001 | .004 | .997 |

$$log\left[ \frac{{bla}_{\mathrm{SHV}}}{16SrRNA} \right]$$

| ***R*** = | .451 | ***R^2^*** = | .203 | ***P*** *=* | .298 |
| --- | --- | --- | --- | --- | --- |
|  |  |  |  |  |  |
|  | **Coefficient (B)** | **Standard**  **Error** | **Standardised Coefficients (*β*)** | ***t*-score** | **Signific.**  **(*p*)** |
| Constant | -4.85 | .98 |  | -4.98 | .000 |
| Chromium, total | -.0031 | .0015 | -.456 | 2.02 | .052 |
| Copper, total | .0097 | .0046 | .524 | 2.11 | .043 |
| Nickel, total | .0012 | .0054 | .053 | .214 | .832 |
| Lead, total | -.0005 | .0006 | -.169 | -.911 | .370 |
| Iron, extractable | .0019 | .0026 | .127 | .735 | .468 |
| pH | .125 | .160 | .136 | .784 | .439 |

$$log\left[ \frac{{bla}_{CTX-M}}{16SrRNA} \right]$$

| ***R*** = | .725 | ***R^2^*** = | .526 | ***P*** *=* | .000 |
| --- | --- | --- | --- | --- | --- |
|  |  |  |  |  |  |
|  | **Coefficient (B)** | **Standard**  **error** | **Standardised Coefficients (*β*)** | ***t*-score** | **Signific.**  **(*p*)** |
| Constant | -5.20 | .72 |  | -7.24 | .000 |
| Chromium, total | .0030 | .0011 | .409 | 2.84 | .007 |
| Copper, total | .0006 | .0030 | .034 | .182 | .856 |
| Nickel, total | -.0045 | .0039 | -.220 | -1.14 | .263 |
| Lead, total | .0004 | .0004 | .133 | 1.04 | .305 |
| Iron, extractable | -.0044 | .0020 | -.269 | -2.20 | .035 |
| pH | .456 | .119 | .469 | 3.82 | .001 |

$$log\left[ \frac{{bla}_{\mathrm{OXA}}}{16SrRNA} \right]$$

| ***R*** = | .539 | ***R^2^*** = | .291 | ***P*** *=* | .043 |
| --- | --- | --- | --- | --- | --- |
|  |  |  |  |  |  |
|  | **Coefficient (B)** | **Standard**  **error** | **Standardised Coefficients (*β*)** | ***t*-score** | **Signific.**  **(*p*)** |
| Constant | -4.54 | .62 |  | -7.37 | .000 |
| Chromium, total | .0008 | .0009 | .164 | .932 | .358 |
| Copper, total | .0032 | .0026 | .280 | 1.22 | .230 |
| Nickel, total | .0005 | .0034 | .037 | .157 | .876 |
| Lead, total | .0000 | .0004 | -.007 | -.043 | .966 |
| Iron, extractable | .0006 | .0017 | .052 | .348 | .730 |
| pH | .210 | .102 | .308 | 2.05 | .048 |

$$log\left[ \frac{erm(C)}{16SrRNA} \right]$$

| ***R*** = | .550 | ***R^2^*** = | .302 | ***P*** *=* | .456 |
| --- | --- | --- | --- | --- | --- |
|  |  |  |  |  |  |
|  | **Coefficient (B)** | **Standard**  **Error** | **Standardised Coefficients (*β*)** | ***t*-score** | **Signific.**  **(*p*)** |
| Constant | -4.30 | 1.33 |  | -3.22 | .006 |
| Chromium, total | .0041 | .0026 | .630 | 1.60 | .131 |
| Copper, total | -.0025 | .0097 | -.083 | -.256 | .801 |
| Nickel, total | -.0185 | .0104 | -.786 | -1.79 | .096 |
| Lead, total | .0004 | .0008 | .176 | .501 | .624 |
| Iron, extractable | .0068 | .0056 | .332 | 1.22 | .244 |
| pH | -.003 | .218 | -.004 | -.014 | .989 |

$$log\left[ \frac{erm(E)}{16SrRNA} \right]$$

| ***R*** = | .522 | ***R^2^*** = | .272 | ***P*** *=* | .096 |
| --- | --- | --- | --- | --- | --- |
|  |  |  |  |  |  |
|  | **Coefficient (B)** | **Standard**  **error** | **Standardised Coefficients (*β*)** | ***t*-score** | **Signific.**  **(*p*)** |
| Constant | -6.29 | 1.22 |  | -5.18 | .000 |
| Chromium, total | .0006 | .0016 | .065 | .339 | .737 |
| Copper, total | -.0022 | .0047 | -.107 | -.459 | .650 |
| Nickel, total | .0029 | .0060 | .119 | .495 | .624 |
| Lead, total | .0004 | .0006 | .096 | .566 | .575 |
| Iron, extractable | -.0052 | .0031 | -.273 | -1.68 | .103 |
| pH | .506 | .204 | .399 | 2.49 | .018 |

$$log\left[ \frac{erm(F)}{16SrRNA} \right]$$

| ***R*** = | .663 | ***R^2^*** = | .440 | ***P*** *=* | .049 |
| --- | --- | --- | --- | --- | --- |
|  |  |  |  |  |  |
|  | **Coefficient (B)** | **Standard**  **error** | **Standardised Coefficients (*β*)** | ***t*-score** | **Signific.**  **(*p*)** |
| Constant | -4.83 | .99 |  | -4.90 | .000 |
| Chromium, total | -.0029 | .0015 | -.478 | -1.90 | .073 |
| Copper, total | .0150 | .0047 | .811 | 3.24 | .004 |
| Nickel, total | -.0011 | .0047 | -.049 | -.224 | .825 |
| Lead, total | -.0002 | .0005 | -.083 | -.415 | .682 |
| Iron, extractable | -.0051 | .0027 | -.323 | -1.87 | .076 |
| pH | .118 | .165 | .127 | .711 | .485 |
