# Supplementary material for: Antibiotic Resistance Gene Abundances Correlate with Metal and Geochemical Conditions in Archived Scottish Soils
Source: PLoS One. 2011 Nov 9;6(11):e27300. doi: 10.1371/journal.pone.0027300 (PMC3212566; doi:10.1371/journal.pone.0027300)
Supplement: Table S6 — One-way ANOVA statistics describing treatment differences in Hartwood and Auchincruive Cu-amended agricultural plots. (DOCX) [file pone.0027300.s007.docx]

**Supplementary Table S6.** One-way *ANOVA* statistics describing treatment differences in Hartwood and Auchincruive Cu-amended agricultural plots.

| **Hartwood Site** |  | Sum of squares | d.f. | Mean squares | F | Sig. |
| --- | --- | --- | --- | --- | --- | --- |
| $\mathbf{log}\left( \frac{\boldsymbol{tet(}\mathbf{M}\boldsymbol{)}}{\boldsymbol{16}\mathbf{S} \mathbf{rRNA}} \right)$ | Between groups  Within groups  Total | 2.789  2.901  5.691 | 4  6  10 | 0.697  0.484 | 1.442 | 0.327 |
| $\mathbf{log}\left( \frac{\boldsymbol{tet(}\mathbf{W}\boldsymbol{)}}{\boldsymbol{16}\mathbf{S} \mathbf{rRNA}} \right)$ | Between groups  Within groups  Total | 1.760  6.830  8.590 | 4  7  11 | 0.440  0.976 | 0.451 | 0.770 |
| $\mathbf{log}\left( \frac{\boldsymbol{bla}_{\mathbf{TEM}}}{\boldsymbol{16}\mathbf{S} \mathbf{rRNA}} \right)$ | Between groups  Within groups  Total | 0.954  0.489  1.443 | 4  7  11 | 0.238  0.070 | 3.411 | 0.075 |
| $\mathbf{log}\left( \frac{\boldsymbol{bla}_{\mathbf{SHV}}}{\boldsymbol{16}\mathbf{S} \mathbf{rRNA}} \right)$ | Between groups  Within groups  Total | 1.238  0.478  1.716 | 4  7  11 | 0.310  0.068 | 4.535 | 0.040 |
| $\mathbf{log}\left( \frac{\boldsymbol{bla}_{\mathbf{CTX}}}{\boldsymbol{16}\mathbf{S} \mathbf{rRNA}} \right)$ | Between groups  Within groups  Total | 1.576  0.746  2.322 | 4  6  10 | 0.394  0.124 | 3.168 | 0.100 |
| $\mathbf{log}\left( \frac{\boldsymbol{erm(}\mathbf{F}\boldsymbol{)}}{\boldsymbol{16}\mathbf{S} \mathbf{rRNA}} \right)$ | Between groups  Within groups  Total | 0.406  1.466  1.872 | 4  6  10 | 0.102  0.244 | 0.416 | 0.792 |

| **Auchincruive Site** |  | Sum of squares | d.f. | Mean squares | F | Sig. |
| --- | --- | --- | --- | --- | --- | --- |
| $\mathbf{log}\left( \frac{\boldsymbol{tet(}\mathbf{M}\boldsymbol{)}}{\boldsymbol{16}\mathbf{S} \mathbf{rRNA}} \right)$ | Between groups  Within groups  Total | 2.252  3.855  6.106 | 4  8  12 | 0.563  0.482 | 1.168 | 0.393 |
| $\mathbf{log}\left( \frac{\boldsymbol{tet(}\mathbf{W}\boldsymbol{)}}{\boldsymbol{16}\mathbf{S} \mathbf{rRNA}} \right)$ | Between groups  Within groups  Total | 1.232  3.323  4.555 | 4  8  12 | 0.308  0.415 | 0.742 | 0.590 |
| $\mathbf{log}\left( \frac{\boldsymbol{bla}_{\mathbf{TEM}}}{\boldsymbol{16}\mathbf{S} \mathbf{rRNA}} \right)$ | Between groups  Within groups  Total | 0.087  0.153  0.240 | 4  8  12 | 0.022  0.019 | 1.147 | 0.401 |
| $\mathbf{log}\left( \frac{\boldsymbol{bla}_{\mathbf{SHV}}}{\boldsymbol{16}\mathbf{S} \mathbf{rRNA}} \right)$ | Between groups  Within groups  Total | 0.150  0.144  0.294 | 4  8  12 | 0.038  0.018 | 2.078 | 0.176 |
| $\mathbf{log}\left( \frac{\boldsymbol{bla}_{\mathbf{CTX}}}{\boldsymbol{16}\mathbf{S} \mathbf{rRNA}} \right)$ | Between groups  Within groups  Total | 0.716  1.138  1.854 | 4  8  12 | 0.179  0.142 | 1.259 | 0.361 |
| $\mathbf{log}\left( \frac{\boldsymbol{erm(}\mathbf{F}\boldsymbol{)}}{\boldsymbol{16}\mathbf{S} \mathbf{rRNA}} \right)$ | Between groups  Within groups  Total | 0.170  1.554  1.724 | 4  8  12 | 0.043  0.194 | 0.219 | 0.920 |
